# Association between maternal periodontal status and ultrasonographic measurement of fetal growth: A longitudinal study

**DOI:** 10.1038/s41598-020-58396-7

**Published:** 2020-01-29

**Authors:** Ayano Taniguchi-Tabata, Noriko Takeuchi, Yoko Uchida, Daisuke Ekuni, Manabu Morita

**Affiliations:** 1 Department of Preventive Dentistry, Okayama University Hospital, Okayama, Japan; 2 Department of Preventive Dentistry, Okayama University Graduate School of Medicine, Dentistry and Pharmaceutical Sciences, Okayama, Japan

**Keywords:** Periodontitis, Risk factors

## Abstract

The aim of this prospective cohort study was to investigate the association between intrauterine fetal growth patterns and periodontal status in pregnant women. Fifty-three pregnant women were recruited. Periodontitis was diagnosed based on probing pocket depth and clinical attachment level. Maternal urinary 8-hydroxy-2’-deoxyguanosine levels and body mass index were recorded. Ultrasonographic measurements of the biparietal diameter (BPD), abdominal circumference (AC), and femur length (FL) were recorded, and estimated fetal weight (EFW) was calculated. In addition, approximation spline curves of BPD, AC, FL, and EFW were obtained throughout the gestation period. Recorded delivery outcomes were gestational age (GA), birth weight and length, sex, placental weight, and umbilical cord length. Forty-four participants (34.1 ± 4.9 years) were analyzed. Mean neonatal birth weight was 2906.0 ± 544.4 g. On multiple regression analysis, birth weight was related with periodontitis (standardized β = −0.21, *P* = 0.001). For EFW and BPD, the curves of the periodontitis group were located lower than those of the non-periodontitis group, with significant differences after 32 weeks and 20 weeks of GA, respectively. In conclusion, periodontal treatment before conception may be recommended and a good periodontal condition in the early stage of pregnancy at the latest is desirable for infant growth.

## Introduction

Low birth weight (LBW) neonates are at higher risk of postnatal mortality and morbidity and lifestyle diseases in adulthood^1–4^. In Japan, birth weight is decreasing each year, although more than 90% of neonates are born during the normal period of gestation (37–41 weeks). In 2016, the average birth weights were 3.05 kg for males and 2.96 kg for females, and 8.3% and 10.5%, respectively, were delivered with an LBW ( < 2500 g)^5^. Ethnicity/race, low socioeconomic status, multiple pregnancy, smoking, alcohol, drug abuse, and microbial infection have been reported as risk factors for LBW^6,7^. Elucidation of the risk factors that affect fetal growth and birth weight is important.

Periodontal disease is thought to be one of the risk factors for LBW^8–10^. Local inflammation induced by periodontal pathogens including lipopolysaccharide (LPS) plays important roles in the pathophysiological processes of LBW or other adverse outcomes^10–14^. Moreover, there are other possible mechanisms through the increase in oxidative stress related to periodontal tissue inflammation^15,16^. Oxidative stress, which results from a disturbance in the balance between the production of reactive oxygen species (ROS) and antioxidant defenses, is reported to be associated with LBW^17,18^. A meta-analysis based on the observational studies concluded that there was evidence of an association between periodontal disease and LBW^19^.

Next, it would be useful to clarify whether periodontal treatment actually prevents LBW. However, some interventional studies reported that treatment of periodontal disease during pregnancy did not prevent adverse pregnancy outcomes^20–23^. A meta-analysis concluded that treatment of periodontal disease during pregnancy had no significant effect in reducing the incidence of LBW. The onset and duration of the periodontal therapy were generally unclear in the previous trials^24^. One of the possible reasons for the reported lack of effect of periodontal treatment might be ineffective timing of the intervention. Thus, further studies are needed to determine the appropriate timing of periodontal treatment to avoid the negative effects of periodontal disease on fetal growth.

Most previous studies focused only on delivery outcomes to see any association between periodontal disease and fetal growth. However, cross-sectional studies based on birth weight could not sufficiently assess fetal/neonatal growth, because the delivery outcome data would be biased by possible growth restriction, such as preterm deliveries associated with maternal complications or iatrogenic preterm deliveries^25^. On the other hand, few studies reported the effect of periodontal disease on longitudinal patterns of intrauterine fetal growth throughout the entire gestational period.

Ultrasonography is the most frequently used tool for assessment of intrauterine fetal growth. Estimated fetal weight (EFW) is calculated using the biparietal diameter (BPD), femur length (FL), and abdominal circumference (AC) measured by ultrasonography. Fetal growth is diagnosed by comparing individual EFW with the standard EFW curve^26,27^. Ultrasonography could attempt to assess the pattern of increased fetal weight, the period when growth restriction happens, and the growth process of every part or organ of the fetal body selectively^28,29^. However, few studies have investigated the correlation between EFW and periodontal disease using ultrasonography. We hypothesized that the effect of periodontal disease on fetal growth appears at a certain time. The aim of this prospective cohort study was to investigate the association between intrauterine fetal growth patterns and periodontal status in pregnant women.

## Results

**Figure 1:**
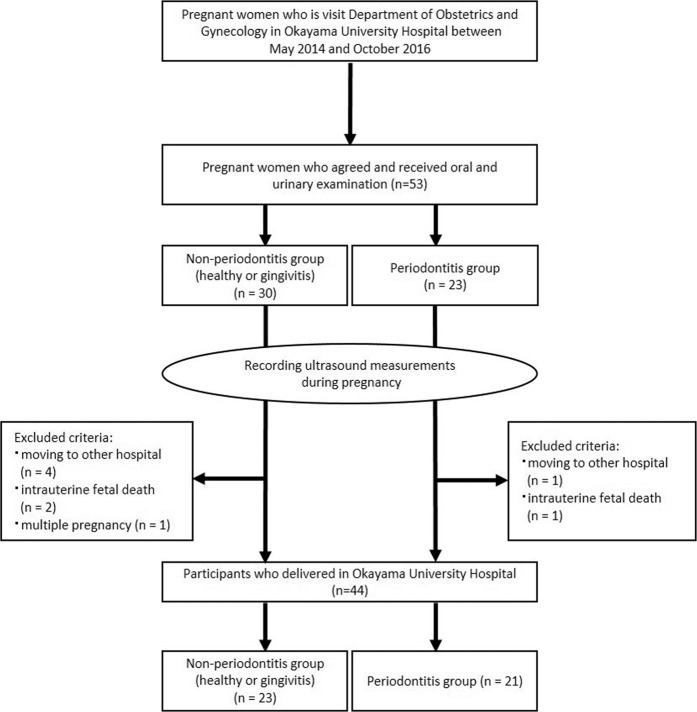
Flow chart of the participants analyzed in the present study.

Figure 1 shows the flow chart for the present study participants. A total of 53 women agreed to participate in the study and underwent oral examinations. Nine participants who did not deliver in the hospital because of moving to another hospital (n = 5), intrauterine fetal death (n = 3), or who had multiple gestations (n = 1) were excluded. Finally, 44 participants (mean age 34.1 ± 4.9 years) were analyzed. Table 1 shows the characteristics of the participants. There were 13 women (29.5%) with baseline disease or pregnancy complications related to decreased birth weight. The details of maternal systemic conditions were as follows: (1) decreased birth weight: antiphospholipid syndrome, heart disease, mood syndrome, and pregnancy complications (threatened premature delivery, threatened abortion, and pregnancy-induced hypertension); (2) no associations with BW: pregnancy with assisted reproductive technology, hypothyroidism, uterine myoma, and protein S deficiency; (3) increased birth weight: type 1 diabetes mellitus and attention deficit hyperactivity disorder. No women smoked or received periodontal treatment during pregnancy. The incidence of preterm birth (gestational age [GA] < 37 weeks) was 11.4% (n = 5). The mean birth weight of the neonates was 2906.0 ± 544.4 g. Four (9.1%) were LBW cases (<2500 g).

**Table 1:** Characteristics of participants.

Maternal age (y)		34.1 ± 4.9^a^
GA at oral examination (weeks)		14.3 ± 2.7
Urinary 8-OHdG (ng/mg CRE)		25.7 ± 13.0
Number of teeth present		28.7 ± 2.1
Mean PPD (mm)		2.4 ± 0.3
Mean CAL (mm)		2.4 ± 0.3
%BOP		19.8 ± 12.7
GA at delivery (weeks)		38.7 ± 2.6
Birth weight (g)		2906.0 ± 544.4
Birth length (cm)		49.6 ± 3.9
Time of delivery	Premature delivery	5 (11.4)^b^
Classification of birth weight	Low birth weight	4 (9.1)
Maternal parity	Multiparous	10 (22.7)
Neonatal sex	Male	18 (40.9)
Placental weight (g)		542.6 ± 101.1
Umbilical cord length (cm)		50.5 ± 9.7
PlI		0.6 ± 0.4
BMI (kg/m^2^)		20.5 ± 4.2
Maternal systemic condition	Decreased BW	13 (29.5)
	Nothing or no associations with BW	29 (65.9)
	Increased BW	2 (4.5)
Periodontal condition	Periodontitis	23 (52.3)

GA gestational age, 8-OHdG 8-hydroxy-2’-deoxyguanosine, PPD probing pocket depth, CAL clinical attachment level, %BOP percentage of bleeding on probing, PlI plaque index, BMI body mass index, BW birth weight.

^a^Mean ± standard deviation.

^b^Number (%).

Table 2 shows the comparison between the non-periodontitis and periodontitis groups. The periodontitis group had lower birth weight than the non-periodontitis group (*P* = 0.016).

**Table 2:** Comparison between the non-periodontitis and periodontitis groups.

		Non-periodontitis	Periodontitis	*P*
		n = 21	n = 23	
Maternal age (y)		34.5 ± 4.2^a^	33.8 ± 5.6	0.645^d^
GA at oral examination (weeks)		13.0 (12.0, 15.0)^b^	14.0 (12.0, 17.0)	0.379^e^
Urinary 8-OHdG (ng/mg CRE)		28.8 (16.9, 40.1)	18.9 (14.9, 24.4)	0.069
Mean PPD (mm)		2.2 ± 0.2	2.5 ± 0.3	< 0.001
Mean CAL (mm)		2.2 ± 0.2	2.6 ± 0.3	< 0.001
%BOP		14.4 (10.6, 27.7)	17.7 (12.0, 24.1)	0.511
GA at delivery (weeks)		39.0 (38.2, 40.0)	39.6 (37.3, 40.4)	0.724
Birth weight (g)		3122.0 (2856.0, 3431.0)	2818.0 (2546.0, 3126.0)	0.016
Birth length (cm)		51.0 (49.8, 52.3)	50.0 (47.5, 52.0)	0.093
Time of delivery	Premature delivery	1 (4.8)^c^	4 (17.4)	0.348^f^
Classification of birth weight	Low birth weight	0 (0)	4 (17.4)	0.109
Maternal parity	Multiparous	6 (28.6)	4 (17.4)	0.481
Neonatal sex	Male	9 (42.9)	9 (39.1)	1.000
Placental weight (g)		568.8 ± 94.8	518.7 ± 102.8	0.101
Umbilical cord length (cm)		52.6 ± 9.0	48.6 ± 10.0	0.172
PlI		0.46 (0.25, 0.59)	0.67 (0.25, 0.96)	0.129
BMI (kg/m^2^)		19.7 ± 4.6	21.3 ± 3.7	0.210
Maternal systemic condition				0.267
	Decreased BW	5 (23.8)	8 (34.8)	
	Nothing or no associations with BW	14 (66.7)	15 (65.2)	
	Increased BW	2 (9.5)	0 (0)	

GA gestational age, 8-OHdG 8-hydroxy-2’-deoxyguanosine, PPD probing pocket depth, CAL clinical attachment level, %BOP percentage of bleeding on probing, PlI plaque index, BMI body mass index, BW birth weight.

^a^Mean ± standard deviation.

^b^Median (25 percentile, 75 percentile).

^c^Number (%).

^d^Non-paired *t-*test.

^e^Mann-Whitney *U* test.

^f^Chi-squared test or Fisher’s exact test.

Birth length, GA at delivery, placental weight, umbilical cord length, and maternal systemic condition had positive correlations with birth weight (all *P* < 0.05). On the other hand, mean probing pocket depth (PPD) and clinical attachment level (CAL) were negatively correlated with birth weight (both *P* < 0.05). No significant association was observed between 8-hydroxy-2’-deoxyguanosine (8-OHdG) concentration and birth weight (Table 3), and between maternal parity and sex or birth weight (Table 4).

**Table 3:** Correlations between birth weight and selected variables.

	Birth weight	
	r	*P*
Birth length (cm)	0.921	< 0.001^a^
Maternal age (y)	0.180	0.242
Urinary 8-OHdG (ng/mg CRE)	0.057	0.715
Mean PPD (mm)	−0.409	0.006
Mean CAL (mm)	−0.394	0.008
%BOP	−0.160	0.300
GA at delivery (week)	0.834	< 0.001
Placental weight (g)	0.791	< 0.001
Umbilical cord length (cm)	0.373	0.013
PlI	−0.149	0.334
BMI (kg/m^2^)	−0.028	0.858
Maternal systemic condition	0.340	0.024^b^

8-OHdG 8-hydroxy-2’-deoxyguanosine, PPD probing pocket depth, CAL clinical attachment level, %BOP percentage of bleeding on probing, GA gestational age, PlI plaque index, BMI body mass index.

^a^Pearson’s correlation analysis.

^b^Spearman’s correlation analysis.

Maternal systemic condition was analyzed using ordinal variables: decreased birth weight 1; nothing or no association with birth weight 2; and increased birth weight 3.

**Table 4:** Comparison of birth weight by related variables.

		Birth weight	
		Mean ± SD	*P*
Maternal parity	Primiparous	2854.5 ± 594.8	0.252
	Multiparous	3081.0 ± 273.7	
Neonatal sex	Female	2810.2 ± 632.8	0.163
	Male	3044.3 ± 355.9	

SD standard deviation.

Non-paired *t-*test.

The results of multiple regression analysis are shown in Table 5. Birth weight was negatively correlated with periodontitis (standardized β = −0.21; *P* = 0.001) and positively correlated with GA at delivery (standardized β = 0.59; *P* < 0.001) and weight of the placenta (standardized β = 0.42; *P* < 0.001).

**Table 5:** Multiple regression analysis of delivery outcomes.

Independent variable	Non-standardized		Standardized β	*P*	95% CI	R^2^
	B	SE				
Maternal age	2.75	6.365	0.02	0.669	−10.22–15.71	0.926
Urinary 8-OHdG	−0.72	2.385	−0.02	0.764	−5.58–4.14	
Multiparous (vs. primiparous)	57.03	66.426	0.04	0.397	−78.27–192.34	
Female neonates (vs. male neonates)	−122.07	66.226	−0.11	0.075	−256.96–12.83	
GA at delivery	125.57	13.274	0.59	< 0.001	98.53–152.60	
Placental weight	2.25	0.344	0.42	< 0.001	1.55–2.95	
Umbilical cord length	−5.34	3.316	−0.09	0.117	−12.10–1.41	
BMI	3.77	6.727	0.03	0.579	−9.93–17.48	
Periodontitis (vs. non-periodontitis)	−228.16	59.232	−0.21	0.001	−384.81 – −107.51	
%BOP	−0.95	2.412	−0.02	0.697	−5.86–3.97	
Maternal systemic condition	46.71	59.905	0.05	0.441	−75.31–168.73	

SE standard error, CI confidence interval, 8-OHdG 8-hydroxy-2’-deoxyguanosine, GA gestational age, BMI body mass index, %BOP percentage of bleeding on probing.

Maternal systemic condition was analyzed using ordinal variables: decreased birth weight 1; nothing or no association with birth weight 2; and increased birth weight 3.

**Figure 2:**
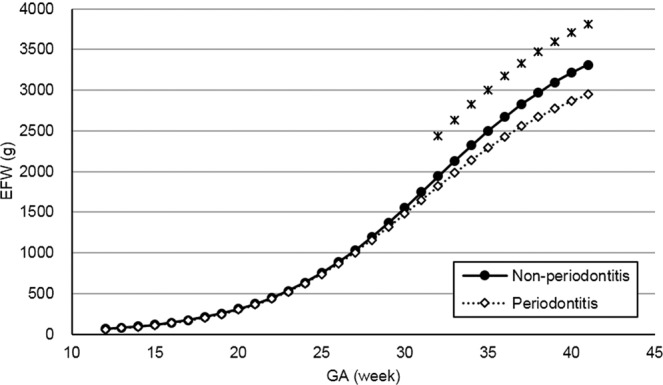
Distribution of estimated fetal weight in the non-periodontitis and periodontitis groups. *EFW* estimated fetal weight, *GA* gestational week. Asterisks show a significant difference at each gestational week (*t*-test, *P* < 0.05).

**Figure 3:**
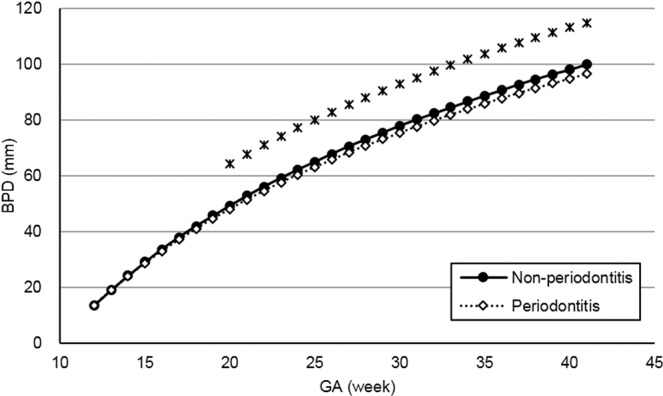
Distribution of biparietal diameter by group (non-periodontitis and periodontitis groups). *BPD* biparietal diameter, *GA* gestational week. Asterisks show a significant difference at each gestational week (*t*-test, *P* < 0.05).

**Figure 4:**
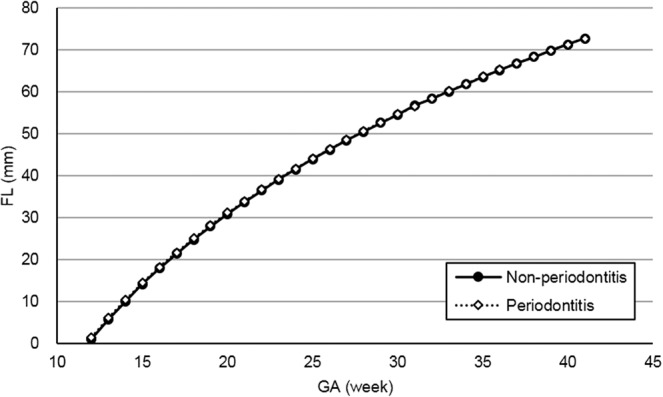
Distribution of femur length by group (non-periodontitis and periodontitis groups). *FL* femur length, *GA* gestational week.

**Figure 5:**
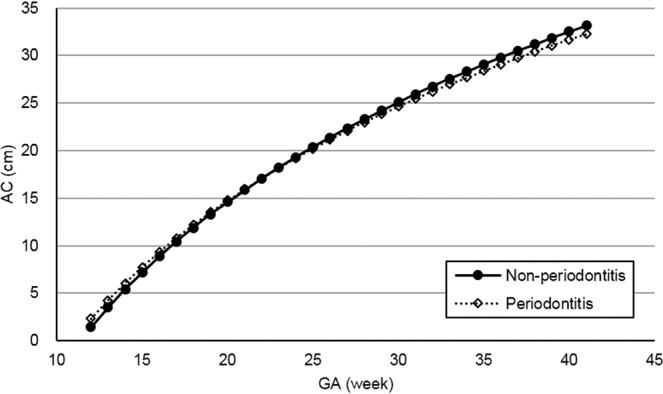
Distribution of abdominal circumference by group (non-periodontitis and periodontitis groups). *AC* abdominal circumference, *GA* gestational week.

Figures 2–5 present the spline curves for EFW, BPD, FL, and AC. For EFW and BPD, the curves of the periodontitis group were located lower than those of the non-periodontitis group throughout the entire gestational period. There were significant differences in EFW and BPD between the non-periodontitis group and the periodontitis group at each week after 32 weeks and 20 weeks, respectively. FL and AC were not significantly different between the two groups in any week throughout the entire gestational period.

## Discussion

The comparison of EFW curves between the non-periodontitis group and the periodontitis group showed that a significant difference in EFW began at 32 weeks of GA. The results suggest that the maternal periodontal condition in the first trimester was associated with fetal growth at a later GA. The previous longitudinal study reported that there was a significant difference in EFW between maternal obesity in the first trimester and normal maternal weight after 32 weeks of GA^25^. These timings were similar to the present study.

There was a significant difference in BPD at 20 weeks and later between the periodontitis and non-periodontitis groups, whereas there were no significant differences in FL and AC. EFW is calculated using BPD, FL, and AC measured by ultrasonography. Thus, the difference in BPD between the two groups may mainly contribute to that in EFW in the present study.

The median of birth weight at delivery was significantly higher in the non-periodontitis group than in the periodontitis group. This finding corresponds to the result of the EFW curve in the present study and is supported by the previous studies^9,10^. On the other hand, some clinical trials reported that maternal periodontal treatment did not prevent LBW^20–23^. Therefore, a systematic review did not recommend routine treatment of periodontal disease during pregnancy for decreasing the risk of LBW^24^. Based on the present results for the EFW and BPD curves, as well as the previous studies, periodontal intervention should be completed in the early stage of pregnancy at the latest, and maintaining a good periodontal condition afterwards is desirable.

There were no significant associations between concentrations of 8-OHdG as a marker of oxidative stress and neonatal birth weight (r = 0.057, *P* = 0.715) or between concentrations of 8-OHdG and maternal periodontal condition (*P* = 0.069). Pregnancy itself can induce oxidative stress because of increasing metabolic activity in the placental mitochondria^30^. When oxidative stress levels are too high, it could interfere with normal placentation. The 8-OHdG levels in urine from pregnant women were associated with decreased birthweight, decreased head circumference, and increased risk of small-for-gestational-age fetus^17,18,31–33^. However, the present findings are inconsistent with these data. The possible reason for the inconsistency may depend on maternal age and 8-OHdG levels. A previous study reported that the median urinary 8-OHdG level of healthy pregnant women (mean maternal age, 25 years) was 5.51 ng/mg CRE at 12 weeks of GA^31^. The 8-OHdG level is correlated with age^34^. In the present study, the mean maternal age was 34 years, and the mean 8-OHdG level was 25.7 ng/mg CRE (mean assay date, 14 weeks of GA). Therefore, it is conceivable that the relatively older age of the participants in the present study obscured the difference in 8-OHdG between the two groups.

There were some limitations of this study. First, all participants were recruited from Okayama University Hospital. This may limit the ability to extrapolate these findings to Japanese women in general. Furthermore, the participants might represent a specific subgroup; only two women were completely healthy. However, the women with systemic diseases received appropriate medical care. Multicenter studies are needed for external validity. Second, the periodontal condition could not be investigated in the second and third trimesters. Finally, previous studies confirmed that systemic inflammation and LPS can affect fetal growth^11–13^. However, these factors could not be investigated in the present study. Further studies are needed to explore the mechanism of the relationship between periodontitis and fetal growth.

In conclusion, after 20 weeks of gestation, BPD was significantly smaller in pregnant women with periodontitis than in those without periodontitis. After 32 weeks of gestation, EFW was significantly smaller. These findings suggest that periodontal treatment before conception may be recommended and a good periodontal condition in the early stage of pregnancy at the latest is desirable for infant growth.

## Methods

### Study population

The present study was a longitudinal observational study. The sample size was calculated by G*Power. The effect size (f^2^) was set at 0.2, alpha error at 0.05, and power (1 – beta) at 0.8 for linear multiple regression (number of predictors was 10), and the minimum sample size was estimated to be 42 participants.

Pregnant women in the first and early second trimester were recruited between May 2014 and October 2016 at the Department of Obstetrics and Gynecology, Okayama University Hospital. Included were those who agreed to participate in the present study and were planning to deliver at the hospital. Exclusion criteria were multiple pregnancy, intrauterine fetal death, and incomplete data on delivery outcome because of unexpected transfer to another hospital. Written, informed consent was obtained from each participant who agreed to participate. The protocol of the present study was approved by the Ethics Committee of Okayama University Graduate School of Medicine, Dentistry and Pharmaceutical Sciences (No. d11002). It was confirmed that all procedures were performed in accordance with the relevant guidelines and regulations. The present study was performed in accordance with the STROBE Statement.

### Oral examination

Five trained dentists carried out the periodontal examination in the first or early second trimester. PPD and CAL were assessed at six sites (mesio-buccal, mid-buccal, disto-buccal, mesio-lingual, mid-lingual, and disto-lingual) for all teeth present with a color-coded probe (Periodontal Probe #5, YDM Co., Tokyo, Japan). Bleeding on probing (BOP) was recorded as present or absent after probing. Mean PPD, mean CAL, and percentage of BOP-positive sites (%BOP) were calculated for each subject. The Plaque Index (PlI)^35^ was also obtained. For PPD and CAL, agreement ± 1 mm was 100% on preliminary calibration.

Participants were divided into two groups according to periodontal status, the non-periodontitis group and the periodontitis group. The presence of 4 or more teeth showing at least one site with PPD ≥ 4 mm and CAL ≥ 3 mm at the same site was diagnosed as periodontitis^10,36^.

### Evaluation of oxidative stress

The urinary 8-OHdG level was measured to assess oxidative stress on the same day as the oral examination, because 8-OHdG has some advantages as a marker. 8-OHdG is present in the extracellular compartment following DNA damage caused by ROS, and it can be noninvasively obtained from urine, as well as serum. 8-OHdG is stable after sample collection, so that it has been used universally to assess oxidative stress^37,38^. The urinary 8-OHdG concentration was determined by an immunochromatographic assay using an automatic analyzer, the ICR-001 (Techno Medica Co., Ltd. Yokohama, Japan)^39–41^. The urine sample was diluted 2 times by distilled water, and 100 μl of the diluted sample were dropped on the testing card. After 5 minutes, the adjusted 8-OHdG concentration was expressed as a ratio to the urinary creatinine concentration (ng/mg CRE).

### Medical examination

Maternal height (cm) and weight (kg) were recorded at the first prenatal visit, as well as pre-pregnancy weight, and body mass index (BMI) was calculated (kg/m^2^). Information on maternal age, parity, baseline disease, complications during pregnancy, and medication use were obtained from the medical records. Maternal systemic conditions were decided based on the maternal baseline diseases and pregnancy complications, and divided into 3 groups by the risk to fetal growth of these abnormalities: 1) decreased birth weight, 2) no associations with BW, and 3) increased birth weight.

Ultrasonographic measurements of the BPD, AC, and FL were recorded by an obstetrician at each visit until delivery. They were blind to the results of the oral examinations. EFW was calculated according to the formula of the Japan Society of Ultrasound in Medicine^26^, which incorporates BPD, AC, and FL:

$${\rm{EFW}}=1.07\times {{\rm{BPD}}}^{3}+3.00\times {10}^{-1}{{\rm{AC}}}^{2}\times {\rm{FL}}.$$

Using the ultrasonographic measurements provided, the time-course regression curves for each participant were computed by a statistical software package. For modeling regression curves, exponential functions were adopted for BPD, FL, and AC, and a logistic curve was adopted for EFW. From the regression curves, the mean fetal measurements were obtained for each GA, and these measurements were splined. Estimated curves were created for the non-periodontitis group and the periodontitis group.

### Delivery outcomes

At the time of delivery, GA, birth weight, birth length, sex, placental weight, and umbilical cord length were recorded by a gynecologist or a nurse.

### Statistical analysis

The distributions of this data were assessed by using Shapiro-Wilk test. After that, non-paired *t*-tests, Mann-Whitney *U* test, and chi-squared tests or Fisher’s exact tests were performed to compare variables between the non-periodontitis and periodontitis groups. Correlations between birth weight and variables were analyzed by Pearson’s or Spearman’s correlation coefficients. Comparisons of birth weight were performed using non-paired *t*-tests in two sets of two groups divided by maternal parity (primipara/multipara) and neonatal sex (male/female), respectively.

Multiple regression analysis was performed to analyze the effects of the following independent variables on birth weight as a dependent variable: maternal age, 8-OHdG level, parity, neonatal sex, GA at delivery, placental weight, umbilical cord length, BMI, periodontal group (non-periodontitis/periodontitis), %BOP, and maternal systemic condition (decreased BW/nothing or no associations with BW/increased BW).

Estimated EFW, BPD, FL, and AC values were compared between the non-periodontitis and periodontitis groups at each GA using non-paired *t*-tests.

All data were analyzed and all estimated curves were created using SPSS version 22 (IBM Japan, Tokyo, Japan) with the level of significance set at *P* < 0.05.
